# Gray matter atrophy in multiple sclerosis despite clinical and lesion stability during natalizumab treatment

**DOI:** 10.1371/journal.pone.0209326

**Published:** 2018-12-21

**Authors:** Fredrika Koskimäki, Jacqueline Bernard, Jeong Yong, Nancy Arndt, Timothy Carroll, Seon-Kyu Lee, Anthony T. Reder, Adil Javed

**Affiliations:** 1 Division of Clinical Neurosciences, Turku University Hospital and University of Turku, Turku, Finland; 2 Department of Neurology, Oregon Health Science University, Portland, Oregon, United States of America; 3 Northwestern University, Biomedical Engineering, Chicago, Illinois, United States of America; 4 Department of Neurology, The University of Chicago, Chicago, Illinois, United States of America; 5 Department of Radiology, The University of Chicago, Chicago, Illinois, United States of America; University at Buffalo, UNITED STATES

## Abstract

**Background:**

Brain volume loss is an important surrogate marker for assessing disability in MS; however, contribution of gray and white matter to the whole brain volume loss needs further examination in the context of specific MS treatment.

**Objectives:**

To examine whole and segmented gray, white, thalamic, and corpus callosum volume loss in stable patients receiving natalizumab for 2–5 years.

**Methods:**

This was a retrospective study of 20 patients undergoing treatment with natalizumab for 24–68 months. Whole brain volume loss was determined with SIENA. Gray and white matter segmentation was done using FAST. Thalamic and corpus callosum volumes were determined using Freesurfer. T1 relaxation values of chronic hypointense lesions (black holes) were determined using a quantitative, in-house developed method to assess lesion evolution.

**Results:**

Over a mean of 36.6 months, median percent brain volume change (PBVC) was -2.0% (IQR 0.99–2.99). There was decline in gray (p = 0.001) but not white matter (p = 0.6), and thalamic (p = 0.01) but not corpus callosum volume (p = 0.09). Gray matter loss correlated with PBVC (Spearman’s r = 0.64, p = 0.003) but not white matter (Spearman’s r = 0.42, p = 0.07). Age significantly influenced whole brain volume loss (p = 0.010, multivariate regression), but disease duration and baseline T2 lesion volume did not. There was no change in T1 relaxation values of lesions or T2 lesion volume over time. All patients remained clinically stable.

**Conclusions:**

These results demonstrate that brain volume loss in MS is primarily driven by gray matter changes and may be independent of clinically effective treatment.

## Introduction

Multiple sclerosis (MS) is a chronic inflammatory and neurodegenerative disease of the central nervous system (CNS). Pathologically, it is characterized by demyelination in the white matter and also substantially in the gray matter, as well as by axonal transection and neuronal loss.[1–3] Loss of tissue in the gray and white matter is associated with cognitive and physical disability. The mechanisms involved and the time course leading to permanent tissue loss continue to be defined, but focal and diffuse inflammation is an important factor in the disease pathology. [4] Current disease modifying treatments (DMTs) for MS target the inflammatory component of the disease with variable effectiveness in curtailing disability. A few longitudinal, prospective, open label studies suggest that some DMTs stabilize certain aspects of MS disease over 5–10 years, [5–7] but none halt all components of MS disease. Due to the difficulties of conducting long-term clinical trials, surrogate markers of disease activity, particularly MRI, are essential for assessing disease status over time.

Quantitative MRI measures of MS disease activity, such as measurement of contrast enhancing T1, T2, and chronic T1 lesions, have been used to predict future relapses, disability progression, and cognitive decline. [8–11] T1 and T2 lesion accumulation over a short time period (2 years) correlate with long-term physical disability.[12, 13] Brain atrophy measurements, both global and regional, assess net tissue damage, since it reflects the sum of demyelination, axonal/neuronal loss, and glial scarring. Brain atrophy reflects current physical and cognitive disability and has prognostic value[14–21]. Regional brain atrophy is a more sensitive and reliable indicator of disease status. Regional gray matter is less prone to pseudoatrophy/artifact effects and certain gray and white matter structures such as the thalamus and corpus callosum show volumetric changes early in the disease course.[22–25]

Brain atrophy occurs in normal aging but is 2–3 fold faster in MS[26] and is present at the earliest stages of MS, in radiologically and clinically isolated syndromes (RIS, CIS).[21, 27, 28]. Reduction in the rate of brain atrophy is an important target of MS treatments. Some DMTs consistently reduce brain atrophy (fingolimod and alemtuzumab) [6, 29, 30] but most others have minimal effects or mixed results. The later may be due to methodological differences for assessing atrophy, mechanism of action of drugs, cohort characteristics, pseudoatrophy, early versus late disease, diurnal brain changes, or pre-study disease status (highly inflammatory, quiescent, or progressive disease activity). Furthermore, the observed atrophy over a given assessment period not only reflects current pathological processes, but is also influenced by prior disease activity and severity and its evolution into the assessment period.

Natalizumab, a specific humanized anti-α4 integrin monoclonal antibody, has a potent anti-inflammatory effects in MS. Natalizumab significantly reduces relapses and disability progression in patients with relapsing-remitting MS.[31–34] In the pivotal phase III trial, AFFIRM, natalizumab reduced brain atrophy only in the second year of therapy, but not in the first year, perhaps due to the pseudoatrophy associated with anti-inflammatory therapies.[35] Regional, global, and white matter atrophy over a longer time period needs further elucidation in patients treated with high efficacy treatments. There is a paucity of information on whether changes in gray or white matter predominantly drive global atrophy during therapy. The objective of this study was to examine global and regional gray and white matter volume loss in clinically stable patients treated with natalizumab for 2–5 years. Clinical and conventional MRI measurements of disease activity such as EDSS and T2 lesion volume changes were included. Quantitative assessment of T1 hypointense lesions over time was also done using a novel technique whereby a T1 relaxation distribution is derived from a T1-weighted scan. With this measure, an increase in T1 relaxation time reflects edema, demyelination, and axonal loss.[36] This assessment of T1 relaxation times in lesions is useful in delineating the fate of lesions over time, showing either deterioration or repair.

## Material and methods

### Patients

This was a retrospective study consisting of patients who had been treated with natalizumab for at least 24 months and up to 68 months. All data were derived from archival patient records including radiological database. Natalizumab infusions were given continuously with standard interval dosing schedule. Clinical assessments including EDSS were performed at routine physician office visits every 4–6 months. Only patients who did not have a contrast-enhancing lesion at baseline and did not have any new MRI activity or relapses during the observation period were included. MRIs were available from patients who were followed longitudinally over a mean of 36.55 months (range 24–68 months). Two time points were examined for atrophy assessments, with 5 out 20 patients having the first assessment scan within 4 weeks prior to natalizumab therapy (median 7 days) and 15/20 patients having the first assessment scan after a minimum of 9 doses of natalizumab (median 11.5 months, range 9–18). Hence, majority of patients had a”run-in” exposure to natalizumab prior to the initial MRI assessment. All MRI and clinical data analysis were done blinded to the patient identity. The study was conducted at the University of Chicago Medical Center and approved by the Institutional Review Board under protocol number 15–1042. This study was performed in accordance with the ethical standards laid down in the 1964 Declaration of Helsinki.

### MRI acquisition and analysis

The MR scans were obtained on a 3T Phillips scanner (Philips Medical Systems, Best, The Netherlands). The protocol was as follows; 3D T1-weighted Turbo Field Echo (3DT1TFE) TR = 8 ms, TE = 3.6 ms, flip angle = 15°, voxel size = 1 x 1 x 1 mm^3^ and FLAIR images TR = 11,000 ms, TE = 125 ms, TI = 2800 ms, both with matrix size = 256 x 256, FOV = 224 x 224 mm. All patients were scanned on the same MRI machine using the same protocol throughout the duration of the study. No hardware or software changes occurred during the study period. Percentage brain volume change was estimated using SIENA.[37, 38] Gray and white matter segmentations were done using FAST[39], which incorporates bias field correction algorithms. Both SIENA and FAST were acquired through FSL library, http://www.fmrib.ox.ac.uk/fsl/fslwiki/. Subcortical segmentation of thalamus and corpus callosum was performed with Freesurfer image analysis suite ((http://surfer.nmr.mgh.harvard.edu/). Volumes of thalamus and corpus callosum were multiplied by the segmented brain-to-estimated total intracranial volume (eTIV) scaling factor to correct for the head size. Prior to all image processing, pre-Gd T1 images were corrected for white matter hypointensities corresponding to T2 lesions on FLAIR images. This lesion in-painting improves the quality of nonlinear registration.[40] The lesion in-painting was done using FreeView, [41] whereby white matter lesions are manually assigned intensities matching the surrounding normal appearing white matter. The final image appears as a normal T1-weighted scan. FLAIR images were used to determine T2 lesion volume using Slicer, https://www.slicer.org/.[42] All volumes are reported in mm3 (x 1000 for conversion to cm3).

As an exploratory measure of T1-hypointensity (chronic black holes) evolution over time, T1 relaxation values for regions of interest were calculated using a novel method. Hypointense lesions seen on T1-weighted scans have been reported to be markers of demyelination, axonal loss, and tissue damage.[43–46] T1-hypointense lesions are strongly correlated with the degree of disability and progression over time.[47, 48] However, the examination of hypointense lesions on T1-weighted scans is problematic, especially when assessing lesion evolution in longitudinal studies. Short TR and TE images are referred to as T1-weighted, and the degree of “weighing” (i.e., shades of gray) can be variable over time and is sequence dependent. The lack of absolute signal intensity confounds both longitudinal analysis of lesion evolution and cross-sectional comparisons among groups, where the degree of demyelination should be evaluated as a metric of disease progression, stabilization, or regression. Hence, exact settings and the same scanner needs to be used to reliably measure the degree of T1 hypointensity over time. The degree of hypointensity on T1-weighted images can also vary as lesions evolve for the better or worse over time. It is also challenging to accurately determine the borders of hypointense lesions for calculating volume or even number. One way of circumventing these issues is to measure T1 relaxation values of the region of interest, which represent the same pathological processes as the T1-weighted lesions. Furthermore, T1 value of the lesions is significantly correlated with disability.[49] Herein, T1 values in different MS lesions and the gray matter were determined to generate quantitative values representing tissue destruction or recovery.

### T1 relaxation map derivation from T1 weighted MRI

A T1 relaxation map of the whole brain was derived from previously acquired T1-weighted spin echo sequences, i.e. 3DT1TFE images described above. The MRI physics of the pulse sequence used to acquire the MR images determined the signal equation and parameters used in the regression analysis to model the relationship between signal intensity and T1. In the case of 3DT1TFE, signal regrowth was modelled as a gradient-recalled echo signal equation with short TE (<< T2):

$$\sqrt{S=k(1-e^{-\frac{TR}{T1}})sin\theta /(1-{cos\theta e}^{-\frac{TR}{T1}})}$$

where *TR* and tip angle *θ* are known and the scanner-specific constant *k* (receiver gain, etc.) is fitted by constraining the equation to the signal intensity and T1 of normal appearing WM and GM. The average signal intensity of NAWM and NAGM were obtained from non-lesion white matter and Heschl gyrus, the later not known to be affected in MS. Reference value of T1 was 810 ms for WM and 1350 ms for GM, as previously reported.[50] Once *k* was found, pixel-by-pixel T1 values were calculated depending on the corresponding pixel signal intensity. The approach described is valid for the 3DT1TFE sequence used in this study; however it can be generalizable to different T1 or T2 images by substituting the appropriate MRI signal equation and tissue reference values.

### Statistical analysis

All statistics were performed using STATA 14.1 (Stata Corp., College Station, TX). Normality of continuous variables was determined by Q-Q plots. Paired Student’s t-test and Spearman’s rho were used to analyze continuous variables. General linear regression, univariate and multivariate, was used to examine the effect of age, race, MS disease duration, natalizumab treatment duration, treatment possession ratio (time on natalizumab/MS disease duration), and baseline T2 lesion volume on whole brain volume loss (PBVC).

## Results

The study consisted of 20 patients with relapsing-remitting multiple sclerosis, of which 19 were female and 1 male. 50% of patients were Caucasian, 45% African-American, and 5% Hispanic. The median age was 39.5 years (interquartile range, IQR 30.5–45) and the median disease duration was 9 years (IQR 6–16 years). Median EDSS at baseline was 2.25 (SD ±1.27) and the median T2 lesion load was 7.42 cm^3^ (IQR 2.32–20.87). The median total time on natalizumab was 32.5 months (IQR 24–46 months). The demographic, clinical, and MRI characteristics of patients at baseline are summarized in Table 1.

**Table 1:** Baseline demographic, clinical, and MRI characteristics of study subjects.

Characteristics	Patients treated with natalizumab
	(n = 20)
Median age, years (IQR)	39.5 (30.5–45)
Sex	
Male	1 (5%)
Female	19 (95%)
Race	
Caucasian	10 (50%)
African-American	9 (45%)
Hispanic	1 (5%)
Median disease duration, years (IQR)	9 (6–16)
Median EDSS (±SD)	2.25±1.27
Median total time on natalizumab, months (IQR)	32.5 (24–46)
Time on natalizumab / Total disease duration (%, mean ±SD)	36.6±18.7
Median T2 lesion load, cm^3^ (IQR)	7.42 (2.32–20.87)

Table 2 shows MRI characteristics at baseline and at follow-up. The median decline in whole brain volume loss over an average of 36.6 months was fairly substantial at -2.00% (IQR 0.99–2.99). Despite this brain atrophy, there was no significant change in EDSS score, T2 lesion volume, and T1 relaxation values. The T1 relaxation value of the lesions and total gray matter was examined in a subset of patients (N = 12) due to the availability of all the necessary original sequence data. Even in this subset of patients, gray matter volume declined significantly (p = 0.01) but not the white matter volume (p = 0.11), despite no significant change in the T1 values of both the gray and white matter.

**Table 2:** Evolution of MRI characteristics over time (mean 36.6 months).

Variable	Baseline	Follow-up
Median PBVC (IQR)	-	2.00 (0.99–2.99)
Mean (±SD) T2 lesion volume, cm^3^	13.64 (15.65)	13.64 (15.41)
Median (±SD) EDSS	2.25 (1.27)	2.25 (1.45)
Mean (±SD) T1 values, ms (n = 12)*		
GM	1240.46±56.44	1315.62±135.29
WM lesion	1041.52±125.82	1068.98±132.66

All comparisons were non-significant per paired t-test. PBVC = percentage of brain volume change, GM = gray matter, WM = white matter.

* Only a subset of patients were examined for T1 relaxation values (n-12).

**Fig 1:**
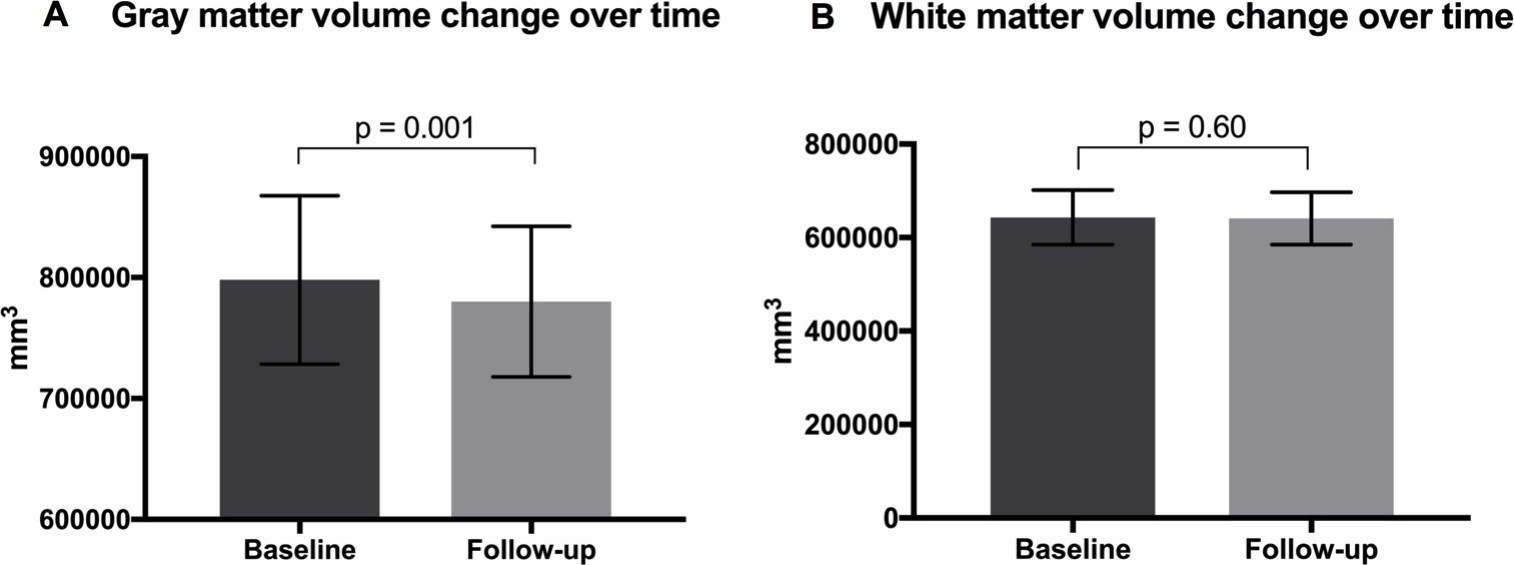
Gray (A) and white (B) matter changes over time (mean 36.6 months).

Fig 1A and 1B show brain segmentation into total gray and white matter volumes and their respective change over time. Total gray matter volume was significant decreased over time compared to the white matter volume (gray matter 798,024.63 ± 15,584.44 SEM mm3 vs. 780,135.80 ± 13,906.12 SEM mm^3^, p = 0.001; white matter 643,247.52 ± 13,030.83 SEM mm^3^ vs. 641,091.86 ± 12,510.08 SEM mm^3^, p = 0.60). Percentage change in gray matter volume over time was -2.24. Even when a smaller cohort of patients in whom MRI was available after a run-in median period of 11.5 months, there was a significant decrease in gray matter volume (gray matter 795,944.62 ± 20,302.09 SEM mm3 vs. 782,322.41 ± 17,687.99 SEM mm^3^, p = 0.024).

**Fig 2:**
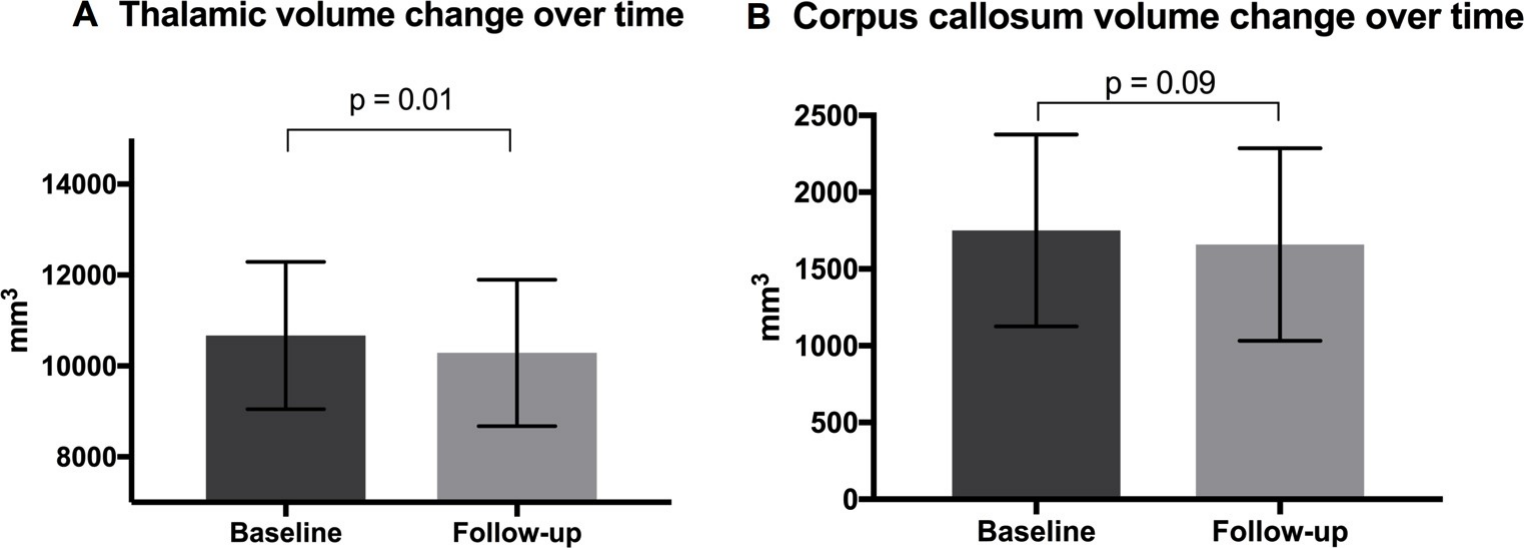
Thalamic (A) and corpus callosum (B) volume changes over time (mean 36.6 months).

Thalamus and corpus callosum were examined next over time (Fig 2). There was a significant atrophy in the thalamus (10,670.98 ± 361.98 SEM mm^3^ vs. 10,286.94 ± 360.27 SEM mm^3^, p = 0.01), but not in the corpus callosum (1,751.24 ± 139.94 SEM mm^3^ vs. 1,659.10 ± 140.30 SEM mm^3^, p = 0.09).

**Fig 3:**
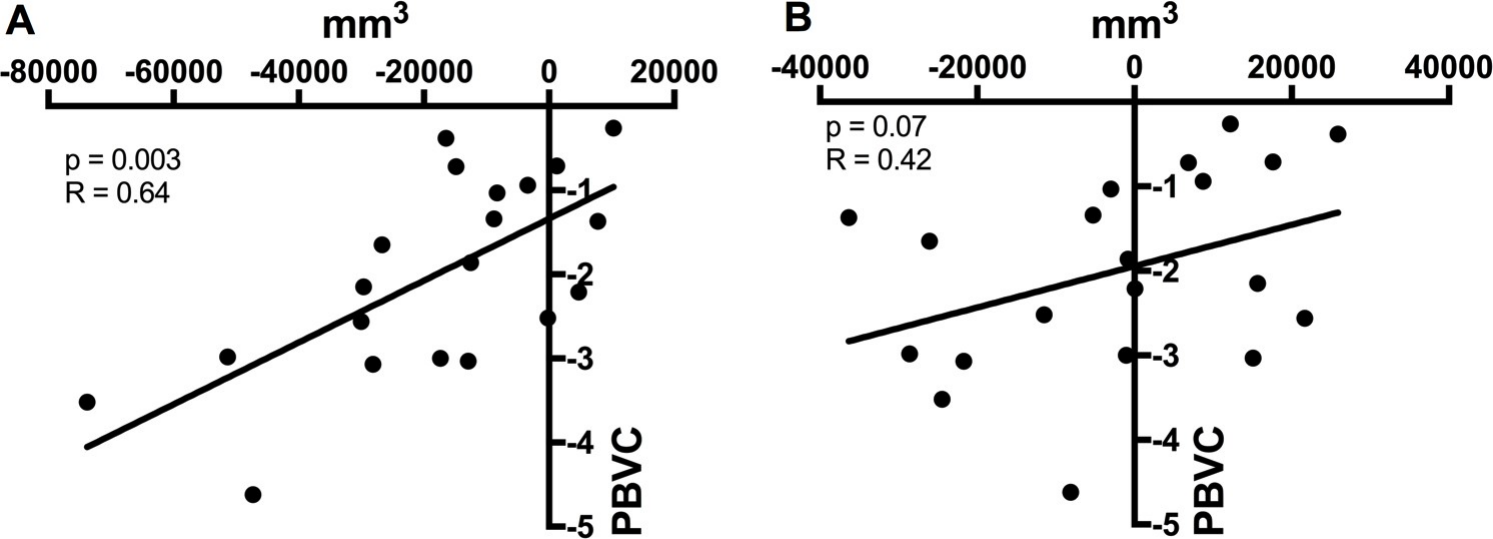
Correlation between PBVC and the change in total gray (A) and white (B) matter volume.

To determine whether brain volume loss over time was driven by the gray or white matter component, PBVC was correlated with the change in the total gray matter (Fig 3A) and the change in the total white matter volume (Fig 3B). There was a significant correlation between the PBVC and the change in gray matter (Spearman’s r = 0.64, p = 0.003) but not between the change in white matter (Spearman’s r = 0.42, p = 0.07).

Univariate regression analysis did not show any significant correlation between MS disease duration, treatment duration, treatment possession, or baseline T2 lesion volume and PBVC (Table 3). However, there was an influence of age on brain volume loss (p = 0.048). In multivariate regression analysis, which included age, MS disease duration, and baseline T2 lesion volume, only age was found to significantly influence PBVC (p = 0.01).

**Table 3:** Univariate and multivariate regression analysis showing the influence of various disease characteristics on brain volume loss (PBVC).

Univariate analysis				
Variable	Coefficient	95% CI	p-value	R^2^
Age	0.049	0.048, 0.096	0.048	0.200
Race	-0.346	-1.46, 0.769	0.523	0.023
Disease duration	-0.006	-0.014, 0.002	0.147	0.113
Treatment duration	-0.018	-0.057, 0.020	0.334	0.052
Treatment possession (%)*	0.007	-0.023, 0.037	0.640	0.012
Baseline T2 lesion volume	-0.026	-0.061, 0.008	0.123	0.127
**Multivariate analysis (age, disease duration, baseline T2 lesion volume)**				
Age	0.062	0.017, 0.106	0.010	0.455

*Time on natalizumab / total disease duration.

## Discussion

MS is a chronic, inflammatory, and more importantly, a neurodegenerative disease. The inflammatory component of the disease has been well studied, but the understanding of the neurodegenerative component and its potential relationship to inflammation remains largely undefined. Over a longer time period, the disease progresses in physical and cognitive domains. Drug treatments slow some aspects of the MS disease process but not all.

Despite clinical disease stability with natalizumab treatment, there was substantial brain volume loss of 2% over a mean period of 36 months. This brain volume loss could not be explained by pseudoatrophy, since the majority of patients had their brain volume assessed using the initial MRI that was acquired at least 9 months post first infusion of natalizumab. Pseudoatrophy phenomena are attributed to resolution of ongoing inflammation. Treatment associated pseudoatrophy is mostly driven by the white matter.[51] Herein, contrast-enhancing lesions at baseline or new T1 contrast-enhancing or T2 lesions were not observed during the study period, precluding the influence of inflammatory disease activity on brain volume loss in all the scans that were analyzed in this study. There was a significant loss of total gray matter but not white matter volume, and based on correlation analysis, total brain volume loss was largely driven by the gray but not the white matter. Furthermore, the thalamus, largely gray matter, demonstrated volume loss over time but the corpus callosum, a white matter structure, did not. Multivariate regression analysis showed that age had significant influence on brain volume loss, but other variables did not, including race, disease duration, treatment duration, treatment possession (time on natalizumab/total disease duration), and baseline T2 lesion volume. Given that age had an influence on brain atrophy over time, the degree of brain atrophy of about 2% could not be explained by the aging phenomena alone in this cohort, since the rate of atrophy in normal aging has been reported to less than 0.4% per year.[52, 53]

Change in the degree of T1 hypointensities, i.e., black holes, was also assessed over time. Rather than measuring change in T1 lesion volume or number, a quantitative and pathologically informative measure was used, T1 relaxation value. This value is reflective of tissue evolution over time, either destructive or reparative. A novel technique is described whereby an already acquired T1 weighted sequence is converted into a T1 relaxation map. T1 relaxation values were derived both for the total gray matter and select lesions in the periventricular regions. Although there was a trend seen in deterioration of T1 relaxation values in the gray matter and white matter lesions, the comparisons did not reach statistical significance (p = 0.14). This could be due to the small sample size. Larger sample size and validations studies are ongoing to further establish the value of this technique. If proven, this technique could have widespread applications, such as retrospective and prospective analysis of MRI data in disease and health.

A key observation of this study was that there was a progressive decline in brain volume despite treatment with natalizumab. This decline was seen in gray matter but not white matter structures. This confirms previous findings that gray and white atrophy occur independently of each other.[16, 54, 55] This study illustrates that although natalizumab could seemingly halt disease progression in the white matter, as determined by conventional MRI methods, gray matter pathology may not be amenable to therapy. Mechanisms that drive gray and white matter changes may be very different. In MS, meningeal inflammation, the presence of follicle-like structures in the meninges, and a unique lymphatic system adjacent to the meninges all point to greater effects on cortical gray matter pathology rather than on white matter. The thalamus, a deep gray matter structure, is affected significantly, perhaps because of its prominent bidirectional connections with the cortical gray matter and because of its close proximity to the choroid plexus, a site of immune cell entry into the CNS.[56] The results of this study suggests that natalizumab may not have substantially halted some of the immune or neurodegenerative mechanisms involved in gray matter atrophy, at least in this cohort of patients. Another possibility is that there may be a slow and steady carry over of prior neurodegenerative decline into the observation period. Given the stabilization of disease in the white matter, it could be that natalizumab has a greater influence in alleviating white matter pathology than that involved in gray matter.

In assessing the effects of a particular drug on brain atrophy, it should be noted that volume loss is a result of several dynamic processes, a balance between destructive and reparative mechanisms with interaction among neurons, axons, oligodendrocytes, astrocytes, microglia, endothelial cells, inflammatory cells, and water distribution. How a particular drug affects a tissue compartment can vary depending on what substrate is most affected by that DMT. Pseudoatrophy is a good example of how some DMTs can initially affect water distribution. Alternately, fingolimod has prominent effects on astrocytes, since they express S1P1 receptors.[57] However, over a longer period of observation time, the interactions among various substrates involved in injury or repair probably reach homeostasis and the net effect is plateau, progression, or continual repair. Furthermore, injury may not have its full effects at the time of occurrence. Prior bouts of inflammation may produce substantial injury, which may not be fully realized until months later as various cells degenerate at different rates, debris is removed, and scarring sets in. Hence, the effects of a particular drug on atrophy may not be fully attributed to that agent due to this carry over effect from prior injury. In terms of MRI analysis of brain volume loss, it is best to set the baseline scan 6–9 months after a relapse, contrast activity, and start of therapy.

Whole or fractionated volumetric changes in patients treated with natalizumab over a longer time period have been previously described. In a subset of patients continuously treated with natalizumab (n = 13), PBVC over a 5-year period was 3.9%; a subset that had non-continuous natalizumab treatment (n = 27), the brain loss was 5%.[58] Both of these subgroups did not have any significant change in their T1 and T2 lesion volume or EDSS over time. Similarly, others have reported a 3% PBVC over a 3-year treatment period with natalizumab treatment.[59] In shorter follow up studies with natalizumab, PBVC has been reported to be reduced by 2.5% (over 18 months, most significant in the first 6 months), and by 1.5% (over 1.5 years).[60, 61]

In other studies examining brain segmentation, gray matter fraction reduction was greatest in the first year (-1.28%) but not statistically significant in the second or third year [55]. The white mater fraction was greatest in the first year (-0.9%) and in the second year (-0.6%), but not statistically significant in the third year. The thalamic volume was significantly reduced in the first and second year but not in the third year. However, the cerebellar gray matter volume loss was seen during all 3 years. The study also showed that physical disability (EDSS) was worse in patients who had lower baseline gray matter fraction and thalamic volume and a greater degree of volume loss at follow-up. Although this study used different methodologies and results are somewhat different than herein, one of the consistencies is that there is still disease progression in terms of gray matter loss over time despite treatment with natalizumab. Another study showed that whole brain volume loss (PBVC) progresses over two years in patients who have contrast enhancing lesions at baseline, showing the influence of baseline inflammation on volume loss for at least two years. [62] A key difference between these studies[55, 62] versus herein is that 47–63% of the patients had contrast-enhancing lesions at baseline, whereas here “stable “patients were studied. Hence, the assessment in earlier studies may be skewed towards stabilization of inflammation by natalizumab rather than preventing the indolent neurodegeneration of the disease, a question raised by the present study.

There are studies that have shown dissimilar findings. A retrospective, one-year study (n = 20) showed no change in whole brain or gray matter volume during natalizumab treatment.[63] A two-year, prospective study (n = 35) showed a significant decrease in cortical atrophy and cortical lesion accumulation during natalizumab treatment.[64] A main explanation for these discordant results is likely to be trial design/observation period and cohort differences, such as age, race, disease duration, prior inflammatory activity, and baseline disease burden.

There are some limitations of the study, the main one being a retrospective study in a small cohort of patients. The clinical measures were not blinded. Brain MRI assessments were made based on available data and not selected *a priori*. Yearly rate of atrophy was not ascertained due to the availability of MRI data. EDSS was measured, but not the more relevant cognitive disability, which is important when examining gray matter changes. Also, a longitudinal, comparative group was not included, such as age/sex matched healthy controls.

Despite these limitations, a salient and consistent finding of this study is that whole brain volume loss over a longer time period is primarily due to gray matter loss, and it continues at a substantial rate even though these patients remain stable physically and per conventional MRI measures. This study also highlights the importance of conducting long-term follow up of patients to capture the neurodegenerative aspect of the disease, particularly as it intersects with brain volume loss due to aging. Clinical trials with longer periods of observation are needed to assess many of these deficiencies, and must include separate examination of gray and white matter. Longer, prospective studies examining a larger sample size and appropriate controls would also clarify the variation in results seen among smaller studies. This study also opens the door for future studies that could employ more sensitive techniques to study neurodegeneration over time such as MTR, DTI, or MRS, and T1 relaxation maps. Also, gray matter changes need to be correlated with cognitive function over a longer time period, an important long-term disability outcome that has been sparsely studied.

## Supporting information

S1 Appendix Additional supporting information for this study. (XLS)
